# Extracellular vesicle‐carried microRNA‐27b derived from mesenchymal stem cells accelerates cutaneous wound healing via E3 ubiquitin ligase ITCH

**DOI:** 10.1111/jcmm.15692

**Published:** 2020-08-26

**Authors:** Shihuan Cheng, Zhiyu Xi, Guang Chen, Kai Liu, Renshi Ma, Chen Zhou

**Affiliations:** ^1^ Department of Rehabilitation the First Hospital of Jilin University Changchun China; ^2^ Department of Vascular Surgery the First Hospital of Jilin University (Eastern Division) Changchun China; ^3^ Personnel Department the First Hospital of Jilin University Changchun China

**Keywords:** extracellular vesicles, IRE1α, ITCH, JUNB, mesenchymal stem cells, microRNA‐27b, wound healing

## Abstract

Mesenchymal stem cells (MSCs) have been highlighted as promising candidate cells in relation to cutaneous wound healing. The current study aimed to investigate whether MSC‐derived extracellular vesicles (EVs) could transfer microRNA‐27b (miR‐27b) to influence cutaneous wound healing. The miR‐27b expression was examined in the established cutaneous wound mouse model, and its correlation with the wound healing rate was evaluated by Pearson's correlation analysis. The identified human umbilical cord MSC‐derived EVs were co‐cultured with human immortal keratinocyte line HaCaT and human skin fibroblasts (HSFs). The mice with cutaneous wound received injections of MSC‐derived EVs. The effects of EVs or miR‐27b loaded on wound healing and cellular functions were analysed via gain‐ and loss‐of‐function approaches in the co‐culture system. Dual‐luciferase reporter gene assay was employed to verify the relationship between miR‐27b and Itchy E3 ubiquitin protein ligase (ITCH). Rescue experiments were conducted to investigate the underlying mechanisms associated with the ITCH/JUNB/inositol‐requiring enzyme 1α (IRE1α) axis. miR‐27b was down‐regulated in the mouse model, with its expression found to be positively correlated with the wound healing rate. Abundant miR‐27b was detected in the MSC‐derived EVs, while EV‐transferred miR‐27b improved cutaneous wound healing in mice and improved proliferation and migration of HaCaT cells and HSFs in vitro. As a target of miR‐27b, ITCH was found to repress cell proliferation and migration. ITCH enhanced the JUNB ubiquitination and degradation, ultimately inhibiting JUNB and IRE1α expressions and restraining wound healing. Collectively, MSC‐derived EVs transferring miR‐27b can promote cutaneous wound healing *via* ITCH/JUNB/IRE1α signalling, providing insight with clinical implications.

## 1 INTRODUCTION

Wound healing represents a complex and dynamic process that influences the quality of life in convalescent, causing high costs for the health system worldwide.^1^ Unfavourable wound healing, particularly in exposed body regions, is not only unappealing visually, but also renders an individual more susceptible to tissue infection, necrosis, poor skin barrier protection and other severe consequences.^2^ Despite advances made in therapeutic wound healing, the complexity of this process remains a notable clinical obstacle worldwide,^3^, ^4^, ^5^ with the mechanism of wound healing yet to be fully understood.^6^ Mesenchymal stem cells (MSCs) represent crucial elements in tissue homeostasis and regeneration owing to their immunomodulatory potential and release of trophic factors that promote healing.^7^ Human umbilical cord–derived MSCs (HUCMSCs) have been identified as promising seeding cells in tissue engineering and clinical applications of regenerative medicine for cutaneous wound healing.^8^ MSC‐derived extracellular vesicles (EVs) are membranous structures loaded with proteins, lipids, carbohydrates and nucleic acids, which play an important role in cell‐cell communication and advances tissue repair.^9^ The abilities of stem cell‐secreted EVs to shape tumour microenvironment, regulate immunomodulation and stimulate endogenous repair processes during tissue damage have been investigated.^10^ Moreover, EVs derived from human umbilical cord‐blood MSCs have been reported to accelerate human skin regeneration.^11^ Importantly, EV‐carried microRNAs (miRNAs or miRs) have been revealed to display potentially therapeutic roles in diabetes‐related impaired wound healing.^12^ It has been highlighted that the transformation of anti‐inflammatory RNAs from stem cells to injury sites mediated by EVs could coordinate the inflammatory responses and immune alleviation, thereby better facilitating the healing processes, which exerts a value and potential for the therapeutic approaches in regenerative medicine.^13^

Accumulating studies have suggested EVs as novel regulators of cell‐to‐cell communication.^14^ EVs derived from MSCs serve as a potential cell‐free therapy for cutaneous regeneration and wound healing.^15^ EVs encompass proteins, messenger RNAs (mRNAs) and miRNAs, which can transfer between cells.^16^ miRNAs have been revealed to associate with the event of pathologic wound healing and hypertrophic scar formation.^17^ Additionally, miR‐27b has been recently identified as a target miRNA that helps to accelerate the process of wound healing in type 2 diabetic mice.^18^ The interaction between miRNAs and their specific target genes has been proposed to be a potential biomarker and therapeutic target of human diseases.^19^, ^20^ The starBase (tarbase.sysu.edu.cn/) predicted binding sites between miR‐27b and the 3’‐untranslated region (UTR) of Itchy E3 ubiquitin protein ligase (ITCH) mRNA. ITCH represents a ubiquitin E3 ligase that regulates protein stability, which has been previously documented to confer protection against the progression of autoimmune disease in human and mice.^21^ Existing literature has suggested that ITCH functions in epidermal homeostasis and remodelling.^22^ Of note, JUNB (a member of the AP‐1 transcription factor family) can define functional and structural integrity of cutaneous epidermo‐pilosebaceous unit.^23^ The JUNB neddylation and JUNB‐dependent transcription are modulated by ITCH.^24^ Moreover, JUNB has been shown to increase the expression of inositol‐requiring enzyme 1α (IRE1α) in osteoblastogenesis,^25^ while IRE1α has been reported to accelerate the process of wound healing in diabetes.^26^ Following the aforementioned exploration of literature, we asserted the hypothesis that miR‐27b from MSC‐derived EVs could influence cutaneous wound healing *via* regulation of ITCH. Hence, our study was designed to validate this hypothesis and to elucidate the ITCH/JUNB/IRE1α axis.

## 2 MATERIALS AND METHODS

### 2.1 Isolation and identification of HUCMSCs

An umbilical cord (about 8 cm in length) from a healthy full‐term newborn was collected and immersed in phosphate‐buffered saline (PBS) containing 1% penicillin/streptomycin (Beyotime, Shanghai, China) and cut into pieces of 2‐3 cm in length. The umbilical cord pieces were subsequently cultured in an inverted T25 cell culture flask containing 2 mL of Dulbecco's modified Eagle's medium/Ham's F‐12 medium (DMEM/F12) (Invitrogen, Carlsbad, CA) with the culture medium renewed every 72 hours. The cells were washed three times with PBS upon reaching approximately 80% cell confluence, detached with 0.25% trypsin (Beyotime, Haimen, China), centrifuged at 1500 r/min for 5 minutes, and passaged at a ratio of 1:2. The HUCMSCs at passage 3‐5 were employed for the isolation of the derived EVs.^27^ The immunohistochemical phenotypic features of HUCMSCs were analysed via flow cytometry. Specifically, HUCMSCs were trypsinized for 2‐4 minutes, washed with calcium and magnesium‐free PBS, and blocked with 10% normal goat serum to avoid non‐specific binding. The cells were then incubated for 30 minutes with fluorescein isothiocyanate (FITC)–labelled monoclonal antibodies against CD14, CD19, CD72, CD34, CD90, CD45, CD105 and HLA‐DR (1:100, BioLegend, San Diego, CA). The cells were subsequently resuspended with 10% normal goat serum (Beijing Solarbio Life Sciences Co., Ltd, Beijing, China) and analysed using CyAn ADP Analyzer (Beckman Coulter, Brea, CA).

### 2.2 Identification of HUCMSCs in vitro

The HUCMSCs were seeded in 6‐well plates at a density of 1 × 10^5^ cells/well. After attachment, the cells were cultured with an osteogenic medium containing DMEM, 0.17 mmol/L vitamin C, 0.5% FBS, 10 mmol/L β‐glycerophosphate, 100 nmol/L dexamethasone and 1% penicillin/streptomycin (Sigma, St Louis, MO) over a period of 21‐28 days with the medium changed every 2 days. Once calcium nodules were visualized under a light microscope (Leica, Frankfurt, Germany), the cells were stained with Alizarin red S staining for further analysis.

Following cell attachment, the cells were cultured with adipogenic differentiation medium containing low‐glucose DMEM containing glutamine, 10% FBS, 1 µmol/L rosiglitazone, 1 µmol/L dexamethasone, 0.5 mmol/L 3‐isobutyl‐1‐methyl‐xanthine, 10 µg/mL insulin, 0.2 mmol/L indomethacin and 1% penicillin/streptomycin. Three days later, the cells were cultured with low‐glucose DMEM supplemented with glutamine, 10% FBS, 1% penicillin/streptomycin, 1 mmol/L rosiglitazone and 10 mg/mL insulin, with the medium renewed every two days. The cell culture duration lasted 21‐28 days with 5% CO_2_ at 37°C. Following detection of lipid droplets, the cells were stained by Oil Red O for further microscopic observation.

HUCMSCs were seeded into 15‐mL centrifuge tubes with a density of about 2 × 10^6^ cells/tube and cultured at 37°C with 5% CO_2_ for 24 hours. After 24 hours, the cells were cultured in chondrogenic medium containing DMEM (4.5 g/L glucose) supplemented with 100 nmol/L dexamethasone, 0.35 mmol/L proline, 0.17 mmol/L vitamin C, 1 mmol/L sodium pyruvate, 1% insulin‐transferrin‐selenium, 10 ng/mL TGFβ‐3 and 1% penicillin/streptomycin (Sigma) for 21‐28 days at 37°C with 5% CO_2_, after which the medium was replaced with a fresh medium on the following day. After the cells had grew into cell spheres with a diameter of 1.5‐2.0 mm, the cells were sliced into sections, stained by Alcian blue, and observed under the light microscope.

### 2.3 Extraction of HUCMSC‐derived EVs

The HUCMSCs were subsequently seeded into a 6‐well plate at a density of 1 × 10^5^ cells/well and cultured in DMEM containing 10% FBS and 1% penicillin/streptomycin (Gibco, Grand Island, NY) until cell confluence reached 80%. The cells were then cultured for 48 hours with Roswell Park Memorial Institute (RPMI) 1640 medium containing EV‐depleted FBS (centrifuged at 100 000 g for 18 hours). The cell supernatant was then centrifuged using Beckman Avanti Centrifuge J‐26XP (Beckman Coulter) for debris and apoptotic body removal. The supernatant was subsequently centrifuged at 110 000 g for 70 minutes (Beckman Optima L‐80 XP Ultracentrifuge with 70Ti rotor), followed by purification by centrifugation at 110 000 g for 70 minutes. All the centrifugations were conducted at 4°C. The precipitates were then resuspended in PBS and sterilized by filtration through a 0.22‐μm filter (Millipore, Darmstadt, Germany).

### 2.4 Identification of HUCMSC‐derived EVs

HUCMSC‐derived EVs were measured using Nanosizer™ instrument ‘dynamic light scattering (DLS)’ (Malvern Instruments, Malvern, UK). The EVs were diluted in 1 mL of PBS and mixed well. The diluted EVs were subsequently injected into the NanoSight NS300 instrument with the particles automatically tracked and sized using Nanoparticle Tracking Analysis (NTA). The morphology of the EVs was observed and analysed using a Hitachi H‐7500 transmission electron microscope (TEM; Hitachi, Tokyo, Japan). More specifically, 10 μL of EVs was placed on formvar carbon‐coated 200‐mesh copper electron microscopy grids, incubated for 5 minutes and stained using standard 1% uranyl acetate for 1 minutes. Prior to TEM observation, the grids were washed three times with PBS and semi‐dried. Finally, Western blot analysis was performed using rabbit antibodies to EV surface markers CD63 (1:2000, ab216130), TSG101 (1:10 000, ab125011), CD81 (1:10 000, ab109201) and endoplasmic reticulum marker Calnexin (1:100 000, ab92573). All the aforementioned antibodies were obtained from Abcam Inc (Cambridge, UK).

### 2.5 Establishment of mouse cutaneous wound model

A total of 90 Kunming male mice aged 9‐12 weeks (weight of 26‐30 g) were recruited in this study. The mice were subsequently anaesthetized by intraperitoneal injection of 50 mg/kg pentobarbital sodium (Sigma) prior to surgical experimentation. Two full‐thickness excisional cutaneous wounds (with a diameter of 12 mm) were made on the dorsum of mice. Following successful model establishment, 30 mice were randomly selected in order to identify the relationship between wound reduction rate and miR‐27b expression. On the fourth day, the wound tissue was photographed with the reduction rate calculated, after which the skin tissue was extracted to determine the expression of miR‐27b. The remaining mice were randomly classified into 4 groups (15 mice per group). Mice in the experimental group were subcutaneously injected with MSCs‐EVs (200 μg dissolved in 100 μL PBS), MSCs‐EVs‐inhibitor‐NC, MSCs‐EVs‐miR‐27b‐inhibitor or an equal volume of PBS at 4 injection sites around the vicinity of the wound. On the 0, 2, 4, 6, and 8 days after operation, these mice were subjected to wound photographing and recording, respectively. All wounds were measured using vernier calliper with the wound area evaluated using Image‐Pro Plus 6 software (Media Cybernetics, Bethesda). The wound reduction rate was calculated using the following formula: wound reduction rate (%) = (A0‐At)/A0 × 100, where A0 was indicative of the initial wound area, and At represented the wound area at 2, 4, 6 and 8 days after the operation. Fifty per cent of the wound healing rate was regarded as the threshold, with a rate above 50% considered to indicate a high healing ability, while <50% was viewed as low healing ability. At 8 days after operation, the mice were killed with their skin samples harvested. The skin samples were subject to histopathological and molecular analyses.

### 2.6 Histopathological analyses

The wound bed and surrounding healthy skin of the mice were collected for further experiments. Haematoxylin‐eosin (HE) staining was performed after which the wounds and surrounding skins (4 mm^2^) were fixed by 4% paraformaldehyde (pH = 7.4), dehydrated by gradient alcohol, embedded in paraffin, and sliced into 4‐μm sections, followed by HE staining (Beyotime) and observation under a light microscope. Based on a previously reported method,^28^ Masson's trichrome staining was conducted to evaluate re‐epithelialization (E%). The tissue sections were dewaxed and stained based on the instructions of the Masson's trichrome staining kit (Beijing Solarbio Life & Sciences Co., Ltd., Beijing, China) to observe collagen formation. Collagen density was evaluated using ImageJ quantitation blue‐stained collagen.^29^

### 2.7 Cell culture

Human immortal keratinocyte line HaCaT (CM‐1252, Mingjing Biology, Shanghai, China) was cultured with 5% CO_2_ at 37°C in DMEM containing 10% FBS and penicillin/streptomycin (all from Gibco). Human skin fibroblasts (HSFs; FH0189, FuHeng Biology, Shanghai, China) were cultured at 37°C with 5% CO_2_ in high‐glucose DMEM containing 10% FBS both from Gibco.

### 2.8 Internalization of MSC‐derived EVs by HSFs and HaCaT cells

Next, in order to determine the internalization of MSC‐derived EVs by HSFs and HaCaT cells, 10 μg of EVs was labelled using green fluorescent dye (PKH67; Sigma) and incubated with 1 × 10^5^ cells at 37°C for 3 hours. The cells were then fixed with 4% paraformaldehyde for 15 minutes followed by the addition of 4',6‐diamidino‐2‐phenylindole (DAPI) (0.5 mg/mL; Invitrogen) for nucleus staining. Finally, green fluorescence was observed under a fluorescence microscope (Leica DMI6000B, Solms, Germany).

Next, to investigate the internalization of MSC‐derived EVs carrying miR‐27b by HSFs and HaCaT cells, Lipofectamine 3000 reagent (L3000001, Invitrogen) was employed to transfect the cy3‐conjugated miR‐27b (GenePharma, Shanghai, China) into MSCs in a serum‐free medium. After 6 hours, the medium was renewed using a medium containing 10% serum without EVs for further incubation for 48 hours. The cell supernatant was then collected, centrifuged and resuspended using PBS, which was then added into the HSFs and HaCaT cells, respectively. Finally, the cells were fixed with 4% paraformaldehyde for 15 minutes and added with DAPI (0.5 mg/mL; Invitrogen) for nucleus staining, followed by fluorescence microscopic observation (Leica DMI6000B, Solms, Germany).

### 2.9 Cell transfection

Cell transfection was performed in accordance with the Lipofectamine 3000 reagent instructions (Invitrogen). The HUCMSCs were delivered with cy3‐miR‐27b or in the presence of GW4869, miR‐27b inhibitor as well as its negative control (NC). The HaCaT cells and HSFs were treated with PBS, MSC‐derived EVs, or EVs derived from the MSCs transfected with miR‐27b inhibitor (MSCs‐EV‐miR‐27b inhibitor). The cells were transfected using plasmids of miR‐27b mimic, ITCH overexpression (OE‐ITCH), OE‐JUNB or their NCs (mimic NC and OE‐NC) alone or in combination. All plasmids were purchased from Guangzhou RiboBio Co., Ltd. (Guangzhou, China).

### 2.10 Cell counting kit‐8 (CCK‐8) assay

CCK‐8 assay (Dojindo, Kyushu Island, Japan) was applied to detect cell proliferation. The cells were seeded in 96‐well plates at a density of 5 × 10^3^ cells/well. At 1‐5 days after treatment, 100 μl of fresh medium containing 10 μl of the CCK‐8 solution was added to each well and incubated at 37°C for 1 hour. A Bio‐Rad 680 microplate reader (Bio‐Rad, Hercules, CA) was used to measure the absorbance at a wavelength of 450 nm. Cell proliferation was expressed as follows: cell proliferation = the absorbance of experimental wells ‐ the absorbance of blank wells.

### 2.11 Scratch test

A total of 1 × 10^5^ HaCaT cells and HSFs were incubated in 6‐well plates until cell fusion had been detected. The monolayer cells were then scraped off using a 200 µL tip whereas exfoliated cells were removed by serum‐free medium washing. The remaining cells were incubated with medium containing 1% FBS and photographed at 0 and 24 hours, respectively. The wound healing area (%) = (A_0_‐A_n_)/ A_0_ × 100, whereby A_0_ was indicative of the area of the initial wound and A_n_ indicated the residual area in wound.

### 2.12 Transwell assay

Cell migration was determined using Transwell chamber (24‐well insert; pore size, 8 µm; Corning Incorporated, Corning, NY). A total of 1 × 10^4^ HaCaT cells and HSFs resuspended in 200 µL of low‐serum (5%) medium were seeded into the apical chamber, with 400 µL complete medium containing 10% FBS subsequently added to the basolateral chamber. After 24 hours, the cells attached to the upper surface of filter membrane were washed while the migrated cells on the lower surface of filter membrane were stained by 0.5% crystal violet (Dingguo Biotechnology Co., Ltd., Changsha, China). Finally, the number of migrated cells was calculated and recorded under the guidance of a microscope (IX83; Olympus, Tokyo, Japan).

### 2.13 Dual‐luciferase reporter gene assay

The human ITCH 3’‐UTR sequence or the mutant sequence of ITCH 3’‐UTR containing the predicted miR‐27b binding sites was inserted into the pGL3 promoter vector (GenScript, Nanjing, China). The HEK293T cells (ATCC, Maryland) were then seeded into 24‐well plates at a density of 5 × 10^5^ cells/well on the day prior to transfection. Next, the cells were co‐transfected with luciferase reporter vectors (0.12 μg) and miR‐27b mimic or mimic NC using Lipofectamine 3000 reagents (Invitrogen).

### 2.14 RNA isolation and quantitation

TRIzol reagents were employed to extract the total RNA (Invitrogen). Next, 1 µg total RNA was reverse transcribed into complementary DNA (cDNA) using the Revert Aid first‐strand cDNA synthesis kit (Fermentas, Life Sciences, Canada). The reverse transcription quantitative polymerase chain reaction (RT‐qPCR) was conducted using a SYBR Premix ExTaqTMII kit in an ABI PRISM® 7900HT System (Takara, Tokyo, Japan). Glyceraldehyde‐3‐phosphate dehydrogenase (GAPDH) was regarded as an internal reference, with the relative mRNA expression determined using the 2^−ΔΔCt^ method. The primer sequences used are depicted in Table 1. The miRNA in EVs was extracted using the SeraMir Exosome RNA Purification Kit (System Biosciences, Mountain View). The cDNA of miRNA was synthesized based on the instructions of TaqMan microRNA assay kit (Applied Biosystems, Foster City). RT‐qPCR was conducted using FastStart Universal SYBR Green Master Mix (Roche, Indianapolis) with the miRNA‐specific forward primer (Sangon Biotech, Shanghai, China) with the reverse primers provided by the TaqMan microRNA assay kit. The level of mRNA was normalized to the U6 gene.

**TABLE 1:** Primer sequences for RT‐qPCR

Target	Primer sequence
hsa‐miR‐27b	F: 5’‐GCTCTAGATTGCCAGGGATTACCACGCAA‐3’
	R: 5’‐CGGGATCCCTAGCATTCCCAGCAGGAGACAG‐3’
mmu‐miR‐27b	F: 5’‐GTGCAGAGCTTAGCTGATTGG‐3’
	R: 5’‐CACTGTGAACAAAGCGGAAAC‐3’
U6	F: 5’‐CTCGCTTCGGCAGCACA‐3’
	R: 5’‐AACGCTTCACGAATTTGCGT‐3’
ITCH	F: 5’‐TGATGATGGCTCCAGATCCAA‐3’
	R: 5’‐GACTCTCCTATTTTCACCAGCTC‐3’
GAPDH	F: 5’‐GGAGCGAGATCCCTCCAAAAT‐3’
	R: 5’‐GGCTGTTGTCATACTTCTCATGG‐3’
JUNB	F: 5’‐CATACACAGCTACGGGATACG‐3’
	R: 5’‐GCTCGGTTTCAGGAGTTTG‐3’
IRE1α	F: 5’‐CGGAATTCATGCCGGCCCGGCGGCTGCTGCTG‐3’
	R: 5’‐AAGCTTTTTGAGCACATGCTTCGCTG‐3’

Abbreviations: RT‐qPCR, reverse transcription quantitative polymerase chain reaction; miR‐27b, microRNA‐27b; ITCH, Itchy E3 ubiquitin protein ligase; GAPDH, glyceraldehyde‐3‐phosphate dehydrogenase; IRE1α, inositol‐requiring enzyme 1α; F, forward, R, reverse.

### 2.15 Western blot analysis

The protein was separated by sodium dodecyl sulfate‐polyacrylamide gel electrophoresis (SDS‐PAGE) and subsequently transferred onto polyvinylidene fluoride membranes (Immobilon‐P, Millipore, Billerica, MA, USA). The membranes were then blocked with 5% milk and Tris‐buffered saline with 0.1% Tween‐20 (TBST) for 1 hours, followed by incubation with primary antibodies at 4°C overnight. The next day, the membranes were re‐probed with horseradish peroxidase (HRP)–labelled secondary antibody at 37°C for 1 hours. The antibodies used included ITCH (A15612, 1:2000, rabbit antibody, abclonal), JUNB (A5290, 1:2000, rabbit antibody, abclonal), IRE1α (ab124945, 1:10 000, rabbit antibody, Abcam), HRP‐labelled anti‐rabbit immunoglobulin G (IgG; 7074, 1:5000, CST) and anti‐mouse IgG (7076, 1:5000, CST). The membranes were then developed using enhanced chemiluminescence reagent (Thermo Fisher Scientific, Waltham, MA, USA) and photographed by ChemiDoc XRS Plus luminescent image analyzer (Bio‐Rad). Image‐Pro Plus 6.0 software was employed for quantitative analysis of protein band intensity, and the relative expression of proteins was expressed as the ratio of the band intensity of target protein to that of GAPDH (A0280, 1:2000, rabbit antibody, abclonal).

### 2.16 Co‐immunoprecipitation (Co‐IP) assay

Following 49 hours of transfection, the cells were collected, lysed by radioimmunoprecipitation assay (RIPA) (Beijing Solarbio Life Sciences Co., Ltd, Beijing, China) containing protease inhibitor cocktail (Roche, Diagnostics GmbH, Mannheim, Germany), and collected. The primary antibodies were incubated with protein A/G‐agarose beads (Santa Cruz Biotechnology Inc, Santa Cruz, CA) for 3‐4 hours at 4°C overnight with the collected precipitates washed at least 3 times in lysis buffer. The precipitates were subsequently added with 2 × SDS loading buffer, boiled for 5 minutes, treated with SDS‐PAGE, and analysed by Western blot analysis. The binding proteins were determined using Co‐IP assay. The antibodies employed for the Co‐IP assay included anti‐Flag (AE005, 1:100, mouse antibody, abclonal) and anti‐HA (AE008, 1:50, mouse antibody, abclonal).

### 2.17 Statistical analysis

Data analysis was performed using SPSS 21.0 statistical software (IBM Corp. Armonk, NY). The measurement data were expressed as mean ± standard deviation. Unpaired data that conformed to normal distribution and homogeneity of variance between two groups were compared by unpaired *t* test. Data among multiple groups were compared by one‐way analysis of variance (ANOVA). The correlation between the two parameters was analysed using Pearson's correlation analysis. A value of *P* < .05 was considered to be reflective of statistical significance.

## 3 RESULTS

### 3.1 miR‐27b exhibits poor expression in the cutaneous wound tissues of mice with low healing ability

**FIGURE 1:**
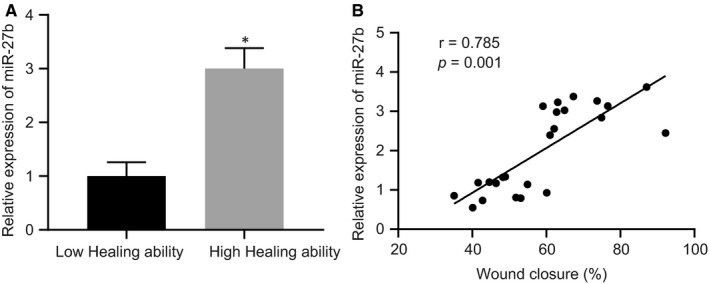
The mice with low healing ability present poor expression of miR‐27b in cutaneous wound tissues. (A) Expression of miR‐27b was determined by RT‐qPCR in the cutaneous wound tissues of mice with high and low wound healing ability, normalized to U6. (B) Pearson correlation of miR‐27b expression with wound reduction rate in mice. *P < .05 compared with mice with low healing ability by unpaired *t* test. Data are shown as mean ± standard deviation of three technical replicates

As per a previously conducted study, miR‐27b is associated with wound healing in type 2 diabetes mellitus.^18^ Next, to determine the relationship between miR‐27b expression and cutaneous wound healing, full‐thickness excisional skin wounds were exerted on the dorsum of mice. On day 4th post‐wounding, the wound area was measured followed by collection of region of wound skin, with RT‐qPCR subsequently performed to determine the expression of miR‐27b. As illustrated in Figure 1A, the expression of miR‐27b was higher in the cutaneous wound tissues of mice with high healing ability than that of the mice with low healing ability. Moreover, Pearson's correlation analysis (Figure 1B) suggested that the wound reduction rate was positively correlated with miR‐27b expression in cutaneous wound tissues. Therefore, miR‐27b expressed poorly in the cutaneous wound tissues of mice with low healing ability and its expression was positively associated with wound healing.

### 3.2 miR‐27b is enriched in HUCMSC‐derived EVs

**FIGURE 2:**
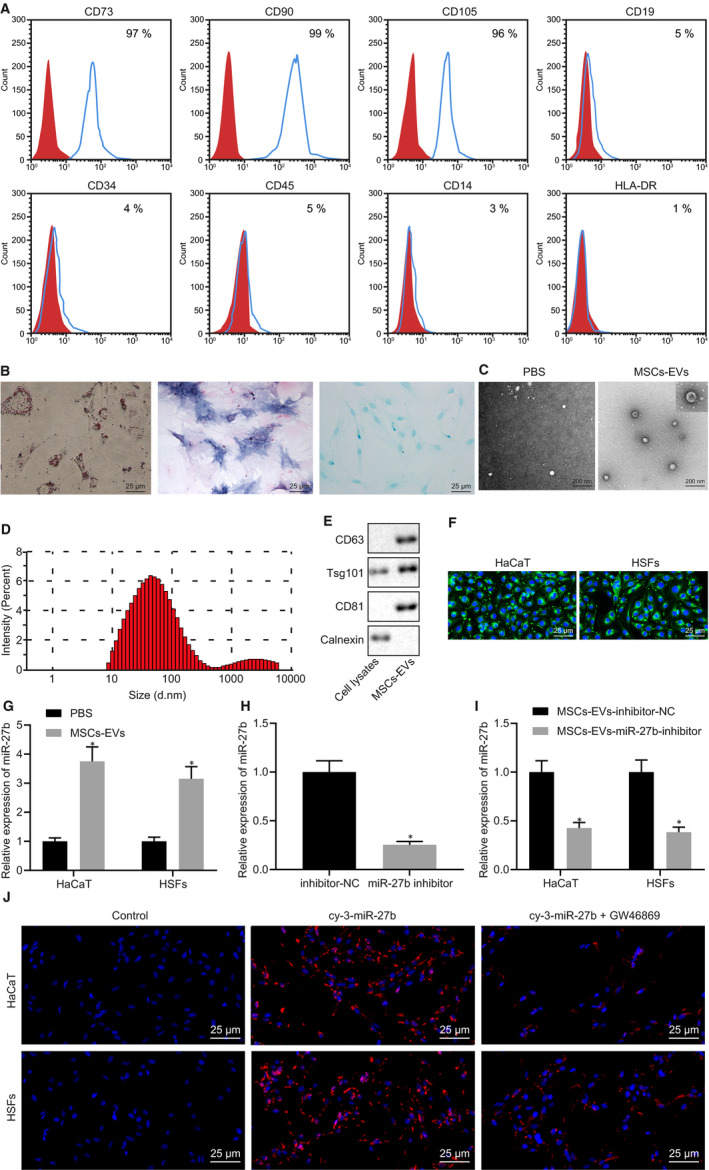
High expression of miR‐27b is found in MSC‐derived EVs. (A) Expression of surface markers of MSCs was detected by flow cytometry. (B) Detection of MSC multi‐differentiation, (i) adipocyte differentiation of cells determined by Oil Red O (scale bar = 25 μm); (ii) osteogenic differentiation of cells examined by Alizarin red S staining (scale bar = 25 μm); (iii) chondrogenic differentiation of cells detected by Alcian blue staining (scale bar = 25 μm). (C) The morphology of MSC‐derived EVs observed under a TEM (scale bar = 200 nm). (D) Size distribution of MSC‐derived EVs was measured by NTA. (E) The expression of CD63 and CD81 in MSC‐derived EVs detected by Western blot analysis. (F) The internalization of MSC‐derived EVs by HaCaT cells and HSFs observed under fluorescence microscope after 4‐h co‐culture of PKH67‐labelled MSC‐derived EVs with HaCaT cells as well as HSFs (scale bar = 25 μm). (G) Expression of miR‐27b was determined by RT‐qPCR in HaCaT cells and HSFs after treatment with MSC‐derived EVs, normalized to U6. *P < .05 compared with cells treated with PBS. (H) Expression of miR‐27b was determined by RT‐qPCR in EVs derived from MSCs transfected with miR‐27b inhibitor, normalized to U6. *P < .05 compared with EVs derived from MSCs transfected with inhibitor‐NC. (I) Expression of miR‐27b was determined by RT‐qPCR in HaCaT cells and HSFs after co‐culture with EVs derived from MSCs transfected with miR‐27b inhibitor, normalized to U6. *P < .05 compared with cells co‐cultured with EVs derived from MSCs transfected with inhibitor‐NC. (J) Delivery of miR‐27b into HaCaT cells and HSFs after co‐culture with MSCs transfected with cy3‐miR‐27b mimic, or MSCs treated with GW4869 (scale bar = 25 μm). Data between the two groups were compared by unpaired *t* test. Data are shown as mean ± standard deviation of three technical replicates

HUCMSC‐derived EVs have been reported to accelerate the rate of healing of scalded cutaneous wounds in rats,^30^ and thus, we speculated that HUCMSC‐derived EVs could improve wound healing by secreting miR‐27b. In order to verify this hypothesis, we examined the expression of miR‐27b in the HUCMSC‐derived EVs. MSCs were isolated from human umbilical cords, and the expression of surface markers of MSCs was determined by flow cytometry. Next, high expression levels of CD73 (97.39%), CD90 (99.34%) and CD105 (96.27%) along with low expression levels of CD19 (5.56%), CD34 (4.83%), CD45 (5.38%), CD14 (3.56%) and HLA‐DR (1.64%) are shown in Figure 2A. Through assessment of the multi‐differentiation abilities, the isolated MSCs were identified to possess osteogenic, adipogenic and chondrogenic differentiation potentials (Figure 2B), highlighting the successful isolation of MSCs. Furthermore, the EVs were isolated from the culture medium of MSCs, and the phenotypic features of MSC‐derived EVs were observed. Under a TEM, the MSC‐derived EVs were observed to be cup‐shaped or cystic‐shaped (Figure 2C), and the results of NTA demonstrated that the diameter of MSC‐derived EVs ranged from 30 nm to 100 nm (Figure 2D). As measured by Western blot analysis, CD63 and CD81 exhibited a greater degree of enrichment in MSC‐derived EVs when compared to the cell debris (Figure 2E). The aforementioned results provided evidence suggesting that the MSC‐derived EVs had been successfully isolated.

Next, to ascertain whether the MSC‐derived EVs could enter into HaCaT cells and HSFs, MSC‐derived EVs were labelled by PKH67 and incubated for 4 hours with HaCaT cells and HSFs, followed by observation under a fluorescence microscope. The results (Figure 2F) demonstrated that both the HaCaT cells and HSFs exhibited green fluorescence, indicating that MSC‐derived EVs entered HaCat cells and HSFs. To explore whether miR‐27b existed in MSC‐derived EVs, HaCaT cells and HSFs were co‐cultured with MSC‐derived EVs, followed by the application of RT‐qPCR to detect the miR‐27b expression in HaCaT cells and HSFs. As depicted in Figure 2G, elevated miR‐27b expression was detected in the HaCaT cells and HSFs co‐cultured with MSC‐derived EVs. Next, to determine whether miR‐27b could enter HaCaT cells and HSFs through MSC‐derived EVs, MSCs were transfected with miR‐27b inhibitor before co‐culture and RT‐qPCR determination. Diminished miR‐27b expression was identified in EVs derived from the MSCs transfected with miR‐27b inhibitor (Figure 2H). Additionally, the HaCaT cells and HSFs were co‐cultured with EVs derived from the MSCs transfected with miR‐27b inhibitor. The results of RT‐qPCR in Figure 2I revealed a decrease in the expression of miR‐27b in the HaCaT cells and HSFs following co‐culture with EVs derived from the MSCs transfected with miR‐27b inhibitor. Additionally, MSCs were transfected with cy3‐miR‐27b mimic and co‐cultured with HaCaT cells and HSFs, after which the MSCs were treated with GW4869. The transfer of miR‐27b into HaCaT cells and HSFs was detected, while GW4869 treatment was found to diminish red fluorescence in the HaCaT cells and HSFs (Figure 2J). Hence, all the above findings led us to conclude that MSC‐derived EVs could transfer miR‐27b into HaCaT cells and HSFs.

### 3.3 MSC‐derived EVs carrying miR‐27b potentiates proliferation and migration of HaCaT cells and HSFs in vitro

**FIGURE 3:**
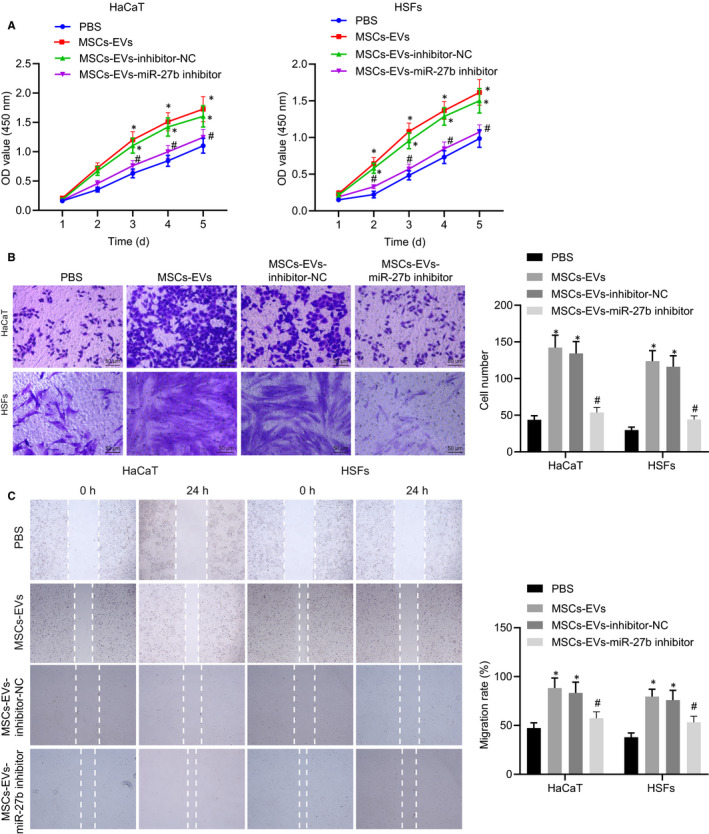
miR‐27b transferred from MSC‐derived EVs promotes proliferation and migration of HaCaT cells and HSFs in vitro. HaCaT cells and HSFs used for following detections were treated with PBS or co‐cultured with EVs derived from MSCs transfected with inhibitor‐NC or miR‐27b‐inhibitor. (A) Proliferation of HaCaT cells and HSFs was detected by CCK‐8 assay. (B) Migration of HaCaT cells and HSFs was assessed by Transwell assay (scale bar = 50 μm). (C) Migration rate of HaCaT cells and HSFs was examined by scratch test. *P < .05 compared with PBS treatment; #*P* < .05 compared with treatment of EVs derived from MSCs transfected with inhibitor‐NC by unpaired *t* test. Data are shown as mean ± standard deviation of three technical replicates

Next, to investigate the finer mechanisms associated with MSC‐derived EVs secreting miR‐27b in accelerating cutaneous wound healing of mice, HaCaT cells and HSFs were selected for in vitro experiments. Similarly, the HaCaT cells and HSFs were co‐cultured with EVs derived from MSCs transfected with inhibitor‐NC or miR‐27b inhibitor. The results of CCK‐8 assay depicted in Figure 3A revealed that proliferation of the HaCaT cells and HSFs was stimulated following treatment with EVs derived from MSCs transfected with inhibitor‐NC relative to PBS treatment; however, their proliferation was markedly inhibited by EVs derived from MSCs transfected with miR‐27b‐inhibitor. Subsequent results from Transwell assay (Figure 3B) and scratch test (Figure 3C) suggested that the co‐culture with EVs derived from MSCs triggered an increase in the migration of HaCaT cells and HSFs, and the co‐culture with EVs derived from MSCs transfected with miR‐27b‐inhibitor led to a marked reduction in cell migration. These findings further proved that MSC‐derived EVs carrying miR‐27b enhanced the proliferation and migration of HaCaT cells and HSFs in vitro.

### 3.4 ITCH is targeted by EV‐carried miR‐27b in HaCaT cells and HSFs

**FIGURE 4:**
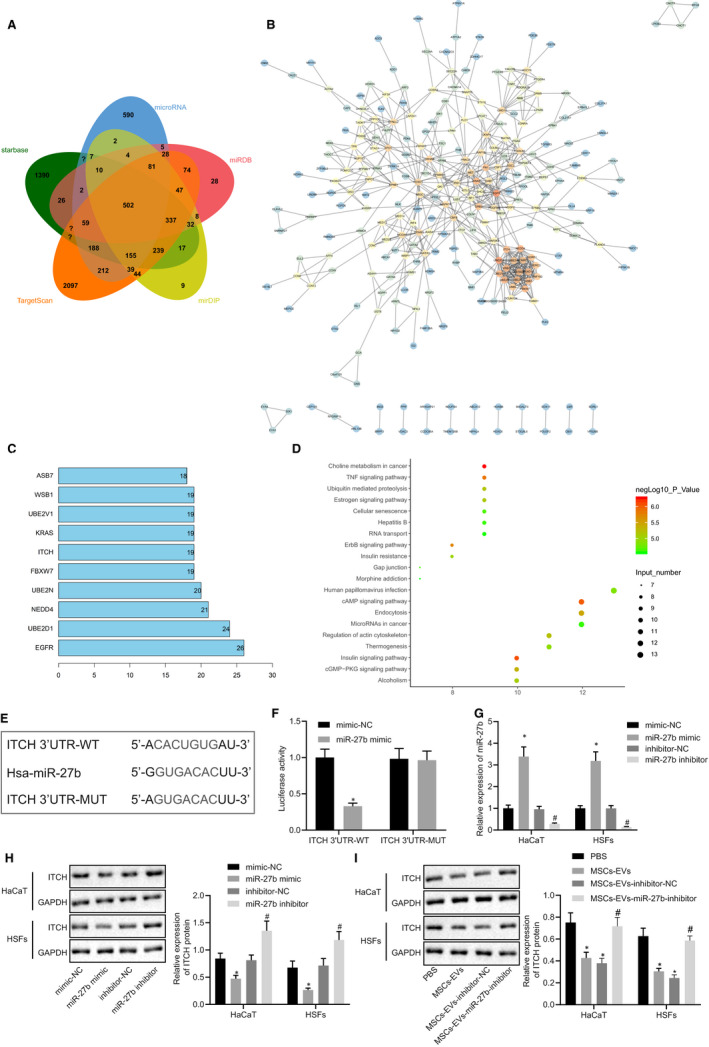
ITCH is directly targeted by miR‐27b in HaCaT cells and HSFs. (A) Predicted downstream target genes of miR‐27b by the starBase, microRNA.org, miRDB, mirDIP and TargetScan databases. The five ellipses in the figure represent the predicted results from the five databases, respectively, and the middle part represents the intersection. (B) Candidate target gene interaction analysis. Each circle in the figure represents a gene, and the line between circles represents the interaction between genes. The more interacted genes of a gene reflected higher core degree, and darker colour. (C) The top 10 genes with the largest core degree value. The abscissa represents the degree value, and the ordinate represents the gene name. (D) KEGG pathway enrichment analysis of candidate target genes. The abscissa represents the gene data in the items, and the ordinate represents the KEGG items. The right histogram represents colour scale. (E) Putative miR‐27b binding sites in the 3’UTR of ITCH mRNA in the starBase database. (F) miR‐27b binding to ITCH was confirmed by dual‐luciferase reporter gene assay in cells; *P < .05 compared with the transfection with mimic‐NC. (G) Expression of miR‐27b was examined by RT‐qPCR in HaCaT cells and HSFs transfected with miR‐27b mimic or inhibitor, normalized to U6; *P < .05 compared with mimic‐NC transfection; #*P* < .05 compared with inhibitor‐NC transfection. (H) Representative Western blots of ITCH protein and its quantitation in HaCaT cells and HSFs transfected with miR‐27b mimic or miR‐27b, normalized to GAPDH. I, Representative Western blots of ITCH protein and its quantitation in HaCaT cells and HSFs treated with PBS and MSC‐derived EVs, normalized to GAPDH; *P < .05 compared with PBS treatment; #*P* < .05 compared with treatment of EVs derived from MSCs transfected with inhibitor‐NC. Data between the two groups were compared by unpaired *t* test. Data are shown as mean ± standard deviation of three technical replicates

Next, we set out to identify the downstream regulatory mechanism of miR‐27b, the starBase (tarbase.sysu.edu.cn/), microRNA.org (http://www.microrna.org/microrna/home.do?tdsourcetag=s_pcqq_aiomsg), miRDB (http://mirdb.org/), mirDIP (http://ophid.utoronto.ca/mirDIP/index.jsp#r) and TargetScan databases (http://www.targetscan.org/vert_71/) were employed to predict the downstream target genes of miR‐27b. Next, the predicted genes from the five databases were intersected (Figure 4A), which revealed 502 candidate target genes (Table S1). The target genes were further subjected to gene interaction analysis using the STRING database (https://string-db.org/), with a gene interaction network map (Figure 4B) subsequently constructed. The top 10 genes with the highest core degree were calculated (Figure 4C). At the same time, the KOBAS3.0 database (http://kobas.cbi.pku.edu.cn/kobas3/?t=1) was explored for KEGG pathway enrichment analysis (Figure 4D). Analysis using protein‐protein interaction (PPI) network and KEGG suggested that ITCH was not only at the core of gene interaction network, but also significantly enriched in the TNF signalling pathway, which has been proposed to be involved in the inter‐vertebral disc disease (IVDD) regulation.^31^

Next, the starBase database used to predict the target gene of miR‐27b with a binding site between miR‐27b and ITCH detected (Figure 4E). Dual‐luciferase reporter gene assay provided evidence indicating that the luciferase activity of ITCH 3’UTR‐WT was notably reduced in HEK293T cells after co‐transfection with miR‐27b mimic, while the luciferase activity of ITCH 3’UTR‐MUT remained unaffected (Figure 4F). RT‐qPCR was performed to analyse the expression of miR‐27b in the HaCaT cells and HSFs following transfection with miR‐27b mimic and miR‐27b inhibitor (Figure 4G), and western blot analysis was employed to measure the ITCH protein expression (Figure 4H). The results obtained revealed that miR‐27b mimic transfection up‐regulated the miR‐27b expression while leading to a down‐regulation in the protein expression of ITCH. However, miR‐27b inhibitor transfection led to a reduction in the expression of miR‐27b and elevated protein expression of ITCH. Additionally, the miR‐27b expression and ITCH protein expression in HaCaT cells and HSFs after co‐culture with MSC‐derived EVs were determined by RT‐qPCR (Figure 2G, I) and western blot analysis (Figure 4I), respectively. The results revealed that MSC‐derived EVs and EVs derived from MSCs transfected with inhibitor‐NC led to down‐regulated protein expression of ITCH in HaCaT cells and HSF, which was restored following miR‐27b inhibitor transfection. Taken together, the findings above suggested that miR‐27b transferred from MSCs *via* EVs and then targeted ITCH in HaCaT cells and HSFs.

### 3.5 The transfer of miR‐27b by MSC‐derived EVs enhances proliferation and migration of HaCaT cells and HSFs through suppression of ITCH in vitro

**FIGURE 5:**
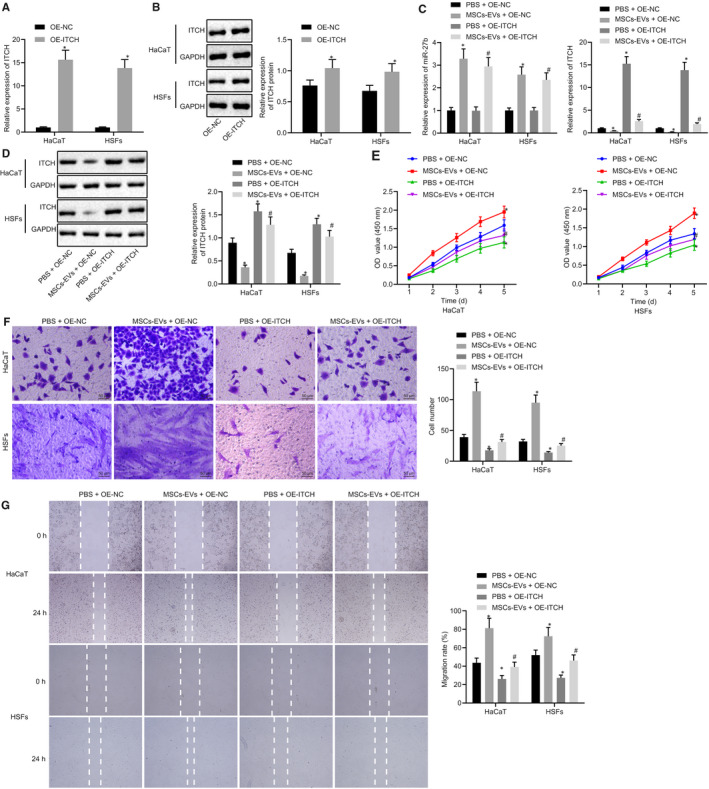
Transfer of miR‐27b *via* MSC‐derived EVs improves proliferation and migration of HaCaT cells and HSFs by repressing ITCH in vitro. (A) mRNA expression of ITCH was examined by RT‐qPCR in HaCaT cells and HSFs after treatments with OE‐ITCH and OE‐NC, normalized to GAPDH. (B) Representative Western blots of ITCH protein and its quantitation in HaCaT cells and HSFs after treatments with OE‐ITCH and OE‐NC, normalized to GAPDH; *P < .05 compared with OE‐NC treatment. The HaCaT cells and HSFs transfected with OE‐NC and OE‐ITCH used for following detections were treated with PBS and MSC‐derived EVs, respectively. (C) Expression of miR‐27b and ITCH was examined by RT‐qPCR in HaCaT cells and HSFs after different treatments, normalized to U6 and GAPDH, respectively. (D) Representative Western blots of ITCH protein and its quantitation in HaCaT cells and HSFs after different treatments, normalized to GAPDH. (E) Proliferation of HaCaT cells and HSFs was examined by CCK‐8 assay after different treatments. (F) Migration of HaCaT cells and HSFs was examined by Transwell assay after different treatments (scale bar = 50 μm). (G) Migration rate of HaCaT cells and HSFs was determined by scratch test after different treatments. *P < .05 compared with HaCaT cells and HSFs transfected with OE‐NC and co‐treated with PBS; #*P* < .05 compared with HaCaT cells and HSFs transfected with OE‐NC and co‐treated with MSC‐derived EVs. Data between the two groups were compared by unpaired *t* test. Data are shown as mean ± standard deviation of three technical replicates

The role of miR‐27b‐mediated inhibition of ITCH in cutaneous wound healing was subsequently investigated. The HaCaT cells and HSFs were transfected with OE‐ITCH and OE‐NC. The results exhibited that the OE‐ITCH transfection increased the ITCH expression in HaCaT cells and HSFs (Figure 5A, B). The HaCaT cells and HSFs transfected with OE‐ITCH were co‐cultured with MSC‐derived EVs. The obtained results (Figure 5C, D) demonstrated that co‐culture with MSC‐derived EVs led to elevated miR‐27b expression but reduced ITCH expression in HaCaT cells and HSFs while transfection of OE‐ITCH enhanced the ITCH expression. Transfection with OE‐ITCH reversed the inhibitory effect of MSC‐derived EVs on ITCH expression in HaCaT cells and HSFs. As shown by the results of CCK‐8 assay in Figure 5E, MSC‐derived EVs enhanced the proliferation of HaCaT cells and HSFs, while the transfection of OE‐ITCH led to a reduction in the proliferation of HaCaT cells and HSFs. The restoration of ITCH counteracted the promotive effect of MSC‐derived EVs on proliferation of HaCaT cells and HSFs. Lastly, Transwell assay (Figure 5F) and scratch test (Figure 5G) revealed that the co‐culture of MSC‐derived EVs led to an enhancement in the migration of HaCaT cells and HSFs while transfection of OE‐ITCH induced a reduction in the migration of HaCaT cells and HSFs. However, overexpression of ITCH was observed to suppress the promotive effect of MSC‐derived EVs on the proliferation and migration of HaCaT cells and HSFs. Collectively, our results suggested that the transfer of miR‐27b from MSC‐derived EVs promoted cell proliferation and migration of HaCaT cells and HSFs by means of inhibiting ITCH in vitro.

### 3.6 MSC‐derived EVs carrying miR‐27b facilitates proliferation and migration of HaCaT cells and HSFs *via* enhancement of the JUNB/IRE1α axis by inhibiting ITCH both in vitro and in vivo

**FIGURE 6:**
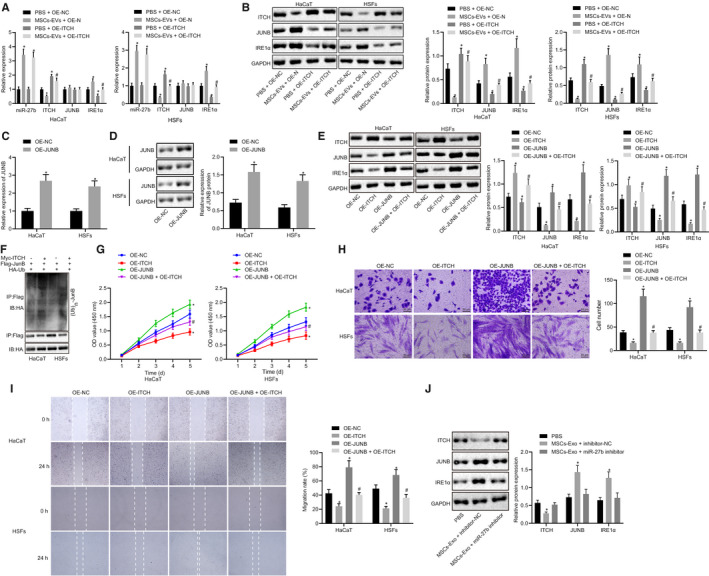
The delivery of mR‐27b from MSC‐derived EVs enhances the proliferation and migration of HaCaT cells and HSFs through JUNB/IRE1α up‐regulation by repressing ITCH both in vitro and in vivo. HaCaT cells and HSFs transfected with OE‐NC or OE‐ITCH used for following detections in panels A and B were co‐treated with PBS and MSC‐derived EVs, respectively. (A) Expression of miR‐27b, ITCH, JUNB and IRE1α was determined by RT‐qPCR in HaCaT cells and HSFs after different treatments, normalized to U6 and GAPDH, respectively. (B) Representative Western blots of ITCH, JUNB and IRE1α proteins and their quantitation in HaCaT cells and HSFs after different treatments, normalized to GAPDH. *P < .05 compared with HaCaT cells and HSFs transfected with OE‐NC and treated with PBS; #*P* < .05 compared with HaCaT cells and HSFs transfected with OE‐NC and treated with MSC‐derived EVs. HaCaT cells and HSFs used for following detections in panels C and D were transfected with OE‐NC or OE‐JUNB. (C) Relative expression of JUNB was determined by RT‐qPCR in cells after different treatments, normalized to GAPDH. (D) Representative Western blots of JUNB protein and its quantitation in cells after different treatments, normalized to GAPDH; *P < .05 compared with OE‐NC transfection. HaCaT cells and HSFs used for following detections in panels (E, G, H and I) were co‐transfected with OE‐ITCH and/or OE‐JUNB. (E) Representative Wester blots of ITCH, JUNB and IRE1α proteins and their quantitation in cells after different treatments, normalized to GAPDH. (F) Effect of OE‐ITCH on JUNB ubiquitination was determined by Co‐IP assay. (G) Proliferation of HaCaT cells and HSFs was examined by CCK‐8 assay after different treatments. (H) Migration of HaCaT cells and HSFs was examined by Transwell assay after different treatments (scale bar = 50 μm). (I) Migration rate of HaCaT cells and HSFs was examined by scratch test after different treatments; *P < .05 compared with OE‐NC treatment; #*P* < .05 compared with OE‐ITCH treatment. (J) Representative Western blots of ITCH, JUNB and IRE1α proteins and their quantitation in skin tissues of wounds in mice after treatments with PBS, MSC‐derived EVs and inhibitor‐NC, or MSC‐derived EVs and miR‐27b inhibitor, normalized to GAPDH. *P < .05 compared with PBS treatment. Data are shown as mean ± standard deviation of three technical replicates. Data between the two groups were compared by unpaired *t* test

Based on aforementioned findings, we hypothesized that miR‐27b promoted wound healing by regulating JUNB and IRE1α *via* ITCH. To testify our hypothesis, HaCaT cells and HSFs transfected with OE‐NC and OE‐ITCH were treated with MSC‐derived EVs and PBS, followed by RT‐qPCR (Figure 6A) and western blot analysis (Figure 6B). The results obtained revealed that co‐culture with MSC‐derived EVs led to reduced expression of ITCH, elevated protein expression of JUNB and IRE1α, whereas no difference in mRNA expression of JUNB was observed in HaCaT cells and HSFs. The overexpression of ITCH triggered a decrease in the protein expression of JUNB and IRE1α while no significant difference was detected in the mRNA expression of JUNB in the HaCaT cells and HSFs. The restoration of ITCH in HaCaT cells and HSFs reversed the increase in protein expression of JUNB and IRE1α induced by the co‐culture with MSC‐derived EVs. Therefore, MSC‐derived EVs carrying miR‐27b mediated the expression of JUNB and IRE1α by suppressing ITCH. Subsequently, HaCaT cells and HSFs were transfected with OE‐JUNB, and the results of RT‐qPCR (Figure 6C) and western blot analysis (Figure 6D) suggested that OE‐JUNB transfection increased the expression of JUNB in HaCaT cells and HSFs. The HaCaT cells and HSFs were then co‐transfected with OE‐ITCH and/or OE‐JUNB. The Western blot analysis (Figure 6E) results illustrated that ITCH overexpression led to a decreased expression of JUNB and IRE1α, while restoration of JUNB reversed the reduction in IRE1α expression in HaCaT cells and HSFs.

Co‐IP assay revealed that OE‐ITCH treatment improved the JUMB ubiquitination and degradation (Figure 6F). Furthermore, the functions of the HaCaT cells and HSFs were evaluated by CCK‐8 assay, Transwell assay and scratch test after co‐transfection with OE‐ITCH and/or OE‐JUNB. The results of CCK‐8 assay (Figure 6G) revealed that ITCH overexpression led to the repression of HaCaT cells and HSFs proliferation, while JUNB overexpression enhanced cell proliferation. Moreover, the overexpression of JUNB reversed the reduction of cell proliferation induced by ITCH. Based on the Transwell assay (Figure 6H) and scratch test (Figure 6I) results, ITCH overexpression was observed to repress cell migration while JUNB overexpression improved cell migration. The concomitant overexpression of ITCH and JUNB led to a greater degree of enhancement in cell migration than ITCH overexpression alone. These results suggested that ITCH suppressed the proliferation and migration of HaCaT cells and HSFs by inhibiting JUNB. Furthermore, the results in Figure 6J demonstrated that treatment with MSC‐derived EVs led to reduced ITCH expression and elevated expression of JUNB and IRE1α in skin tissues of wounds, which was counteracted by inhibition of miR‐27b. Thus, a conclusion could be drawn that MSC‐derived EVs carrying miR‐27b up‐regulated JUNB and IRE1α expressions by suppressing ITCH, ultimately enhancing the proliferation and migration of HaCaT cells and HSFs in vitro and in vivo.

### 3.7 MSC‐derived EVs carrying miR‐27b enhances epidermal re‐epithelialization and collagen fibre proliferation, thus advancing cutaneous wound healing in vivo

**FIGURE 7:**
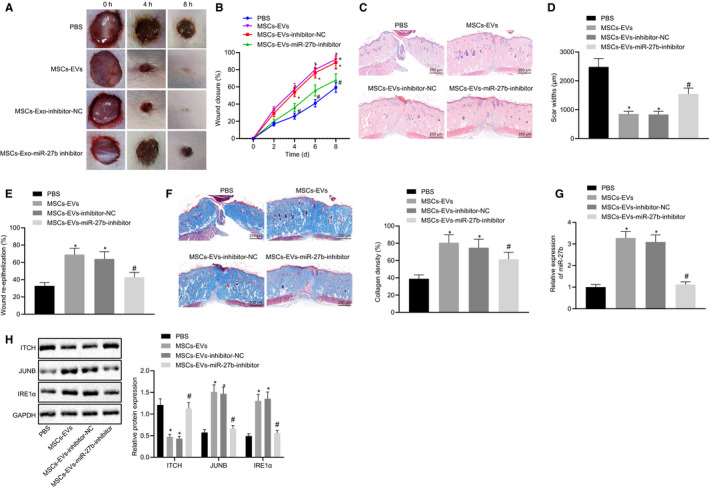
MSC‐derived EVs mitigate epidermal re‐epithelialization and collagen fibre proliferation in wounds by delivering miR‐27b in vivo. The mice used for following detections were treated with PBS, EVs derived from MSCs transfected with inhibitor‐NC or EVs derived from MSCs transfected with miR‐27b‐inhibitor. (A) Size of wounds in mice at the 0, 2, 4, 6 and 8 d after operation. (B) Healing rate of wounds. (C) Pathological changes of cutaneous wound tissues were observed by HE staining in mice at 8 d after operation (scale bar = 250 μm). (D) Quantified scar width in the wound of mice. (E) Wound re‐epithelialization in mice. (F) Masson‐stained collagen in wound tissues of mice (scale bar = 250 μm). (G) miR‐27b expression was determined by RT‐qPCR in cutaneous wound tissues of mice at the 8 d after operation, normalized to U6. (H) Representative Western blots of ITCH, JUNB and IRE1α proteins and their quantitation in cutaneous wound tissues of mice, normalized to GAPDH. *P < .05 compared with PBS treatment; #*P* < .05 compared with EVs derived from MSCs transfected with inhibitor‐NC by unpaired *t* test. The data above were measurement data presented as mean ± standard deviation. n = 15 for mice following each treatment

In order to further elucidate the effects associated with MSC‐derived EVs carrying miR‐27b on cutaneous wound healing of mice, full‐thickness excisional skin wounds were made on the dorsum of the mice. The MSCs were transfected with miR‐27b inhibitor and their derived EVs were isolated, which were subsequently injected subcutaneously into the skin around the wounds of the mice. On day 0, 2, 4, 6 and 8 following the operation, wound photographing and recording were conducted (Figure 7A) with the wound healing rate calculated (Figure 7B). The results revealed that compared with PBS treatment, the treatment of EVs derived from MSCs accelerated the cutaneous wound healing. Furthermore, treatment with EVs derived from MSCs transfected with miR‐27b inhibitor retarded the cutaneous wound healing of mice compared to treatment with EVs derived from MSCs transfected with inhibitor‐NC. HE staining was conducted to analyse the pathological changes of cutaneous wound tissues in mice on the 8th day post operation. The results (Figure 7C) revealed that treatment with EVs derived from MSCs transfected with inhibitor‐NC led to the formation of more new epidermis and dermis compared with PBS treatment. As illustrated in Figure 7D‐E, the results of the scar width and re‐epithelialization provided further verification that treatment with EVs derived from MSCs transfected with inhibitor‐NC enhanced the epidermal re‐epithelialization and reduced scar formation of wounds, while inhibition of miR‐27b in EVs derived from MSCs led to an increase in scar width along with a reduced re‐epithelialization. Analysis of Masson staining (Figure 7F) revealed that treatment of EVs derived from MSCs transfected with inhibitor‐NC induced abundant wavy collagen fibre in wounds, while inhibition of miR‐27b in EVs derived from MSCs led to reduced wavy collagen fibre. Based on the aforementioned findings, we concluded that the inhibition of miR‐27b in MSC‐derived EVs reversed the promotive effect of MSC‐derived EVs on epidermal re‐epithelialization and collagen fibre proliferation in wounds. Furthermore, cutaneous wound tissues of mice led to an increased expression of miR‐27b, JUNB and IRE1α yet decreased ITCH expression in the presence of MSC‐derived EVs and EVs from MSCs transfected with inhibitor‐NC, on the contrary, with a set of contrasting results identified in the absence of miR‐27b in MSC‐derived EVs. Collectively, MSC‐derived EVs could up‐regulate miR‐27b expression and consequently inhibit ITCH expression while elevating the expression of JUNB and IRE1α, ultimately accelerating wound healing in vivo.

## 4 DISCUSSION

Cutaneous wound healing represents a dynamic intricate process with multiple mutually overlapping phases.^32^ Unfavourable wound healing poses a significant threat to public health with an urgent need for fresh novel targets aimed at managing and treating the complications of unfavourable wound healing outcomes.^33^, ^34^ The ability of MSCs to enhance wound healing has been highlighted in existing literature.^35^ Hence, we hypothesized that the transfer of miR‐27b by MSC‐derived EVs could play a critical role in cutaneous wound healing via the regulation of downstream factors. Our findings from the co‐culture experiments MSC‐derived EVs delivering miR‐27b could potentially accelerate the process of cutaneous wound healing through the up‐regulation of JUNB and IRE1α *via* inhibition of ITCH.

Our initial observations revealed that miR‐27b was poorly expressed in the cutaneous wounds of mice with low healing ability. A previous study reported that miR‐27b is down‐regulated in burned skin.^36^ Hence, we set out to further investigate the up‐regulation of miR‐27b in MSC‐derived EVs. MSC transplantation has been previously demonstrated to improve the M2 polarization of macrophages and subsequently enhance wound healing by transferring EV‐derived miR‐223.^37^ Notably, platelet‐rich plasma‐derived EVs have been noted to improve the re‐epithelialization of chronic cutaneous wounds in rat models of diabetes mellitus, thus enhancing cutaneous healing,^38^ while EVs secreted from bone marrow–derived multipotent mesenchymal stromal cells have also been shown to facilitate a more uniform alignment and compactness of collagen fibres in the process of tendon healing.^39^ The current study mainly suggested that MSC‐derived EVs carrying miR‐27b enhanced epithelialization and collagen fibre proliferation in wounds. Enhanced epithelialization and collagen fibre proliferation have been suggested to be indicators of superior wound healing.^40^, ^41^ Hence, MSC‐derived EVs carrying miR‐27b improved wound healing of mice.

A notable findings of our in vitro experiments demonstrated that the MSC‐derived EVs carrying miR‐27b promoted the proliferation and migration of HaCaT cells and HSFs. Furthermore, platelet‐rich plasma‐derived EVs accelerate the proliferation and migration of endothelial cells and fibroblasts in the wound healing process.^38^ The secretion of EVs from keratinocytes may potentially improve wound healing by promoting the proliferative and migrating capacities of endothelial cells and fibroblasts.^42^ Similarly, EVs have been reported to transfer the miR‐21 expression from melanocytes into keratinocytes along with stimulating their proliferation and migration which ultimately contributes to protection against ultraviolet (UV) damage.^43^ During the current study, ITCH was identified as a target gene of miR‐27b. Accordingly, miR‐27b has been demonstrated to bind to target genes such as VEGFC, FRS3, FGF1 and HYOU1, exhibiting a regulatory role in a wide array of processes associated with wound healing.^44^ More recently, evidence has been presented suggesting enhanced wound healing in E3 ligase ITCH knockout mice.^22^ Intriguingly, the rescue experiments preformed in the present study demonstrated that ITCH reversed the promotive effects of MSC‐derived EVs carrying miR‐27b on proliferation and migration of HaCaT cells and HSFs. Thus, we asserted the notion that MSC‐derived EVs carrying miR‐27b could facilitate proliferation and migration of HaCaT cells and HSFs by inhibiting ITCH.

ITCH has also been highlighted to exert a crucial effect on posttranslational modification through ubiquitin proteasomal protein degradation.^45^ Our results illustrated that ITCH degraded JUNB through the ubiquitination and reduction of JUNB expression. ITCH depletion can enhance the cutaneous wound healing, and JUNB has been emphasized in previous reports as a crucial ITCH’s substrate associated with epidermal development and homeostasis.^22^ JUNB also elevates the expression of IRE1α in osteoblastogenesis,^25^ and IRE1α promotes the wound healing in diabetes.^26^ Furthermore, IRE1α overexpression in bone marrow–derived progenitor cells has been demonstrated to accelerate wound healing in diabetic settings.^46^ Our study provided evidence to support the observation that EV‐encapsulated miR‐27b derived from MSCs confers promotion of cutaneous wound healing through the regulation of ITCH/JUNB/IRE1α.

**FIGURE 8:**
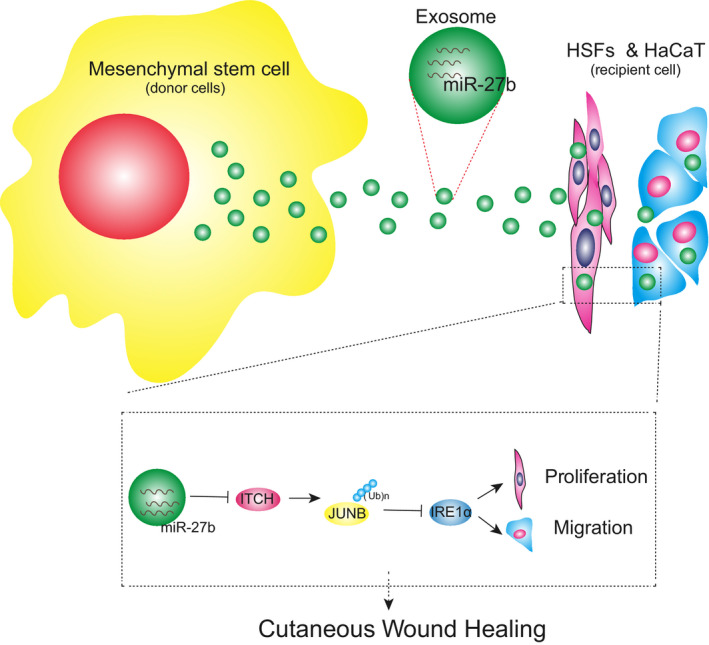
The molecular mechanism of MSC‐derived EVs carrying miR‐27b in affecting cutaneous wound healing through the ITCH/JUNB/IRE1α axis. MSC‐derived EVs carrying miR‐27b suppressed the ITCH expression in HaCaT cells and HSFs, and consequently enhanced the expression of JUNB, and IRE1α, thus improving the proliferation and migration of HaCaT cells and HSFs and accelerating the cutaneous wound healing in mice

Collectively, the key findings of the current study suggest that EVs derived from MSCs transport miR‐27b into HaCaT cells and HSFs to promote cutaneous wound healing. Our results suggested that miR‐27b may repress the expression of ITCH while elevating the expression of JUNB and IRE1α to strengthen the proliferation and migration of HaCaT cells and HSFs, through which EVs derived from MSCs improved the cutaneous wound healing in mice (Figure 8). Thus, EV‐carried miR‐27b should be considered as a promising therapeutic target for wound healing. However, the research is still at the preclinical stage. A transition of this finding to clinical application warrants further investigation with the specific mechanism requiring further elucidation in future studies.

## CONFLICT OF INTEREST

The authors declare that they have no conflict of interest.

## AUTHOR CONTRIBUTION

**Shihuan Cheng:** Investigation (equal); Software (equal); Writing‐original draft (equal); Writing‐review & editing (equal). **ZhiYu Xi:** Formal analysis (equal); Methodology (equal); Writing‐original draft (equal); Writing‐review & editing (equal). **Guang Chen:** Formal analysis (equal); Writing‐original draft (equal); Writing‐review & editing (equal). **Kai Liu:** Data curation (equal); Investigation (equal); Writing‐review & editing (equal). **Renshi Ma:** Supervision (equal); Validation (equal); Visualization (equal); Writing‐review & editing (equal). **Chen Zhou:** Project administration (equal); Resources (equal); Validation (equal); Writing‐review & editing (equal).

## ETHICAL APPROVAL

The collection of umbilical cord samples was approved by the Ethics Committee of the First Hospital of Jilin University (ethical approval date: 2019.02.16; ethical approval number: 201 902 005). All human body–related experiments were performed in strict accordance with the *Declaration of Helsinki*. All participants signed informed consent documentation prior to sample collection. The animal use and experimental procedures were approved by the Animal Ethics Committee of the First Hospital of Jilin University (ethical approval date: 2019.08.31; ethical approval number: 201 908 127). Extensive efforts were made to ensure minimal suffering of the included animals.

## Supporting information

Table S1

## Data Availability

The data sets generated and/or analysed during the current study are available from the corresponding author on reasonable request.
